# A new approach for assessment of clubfoot treatment using non-radiological scanner coupling finite element

**DOI:** 10.3389/fped.2026.1777412

**Published:** 2026-02-09

**Authors:** Shoushen Liu, Dongfang Zhang, Xiao Lv, Xinjie Huan, Fujiang Li

**Affiliations:** Department of Adolescent Sports Medicine, Qingdao Municipal Hospital, Qingdao, China

**Keywords:** 3D model, clubfoot, finite element, non-radiological scanner, Ponseti

## Abstract

**Introduction:**

To develop a new approach for clubfoot treatment assessment using a non-radiological scanner and finite element method (FEM) simulation techniques, which could eliminate the 8% risk of x-ray exposure, as well as evaluate the strain at the Achilles tendon.

**Methods:**

The geometry of the clubfoot was acquired by using an optical-light-based scanner. The 3D model was generated from the geometry and exported to finite element simulation software. The contact and large deformation model was established to 13 simulate the angle correction of the Ponseti therapy. The strain and pseudo strain 14 rate were calculated from the simulation results and analysed for assessment.

**Results:**

The simulated stress distribution showed the stress concentration around the Achilles tendon, which coincides with the actual situation. The strain and pseudo strain rate were analysed with the correction angle, which matches the three-stage casting of Ponseti therapy. The result suggested the intermediate angle correction casting was 40% and 87% of the correction process. The recommended correction angles for coronal, axial, and sagittal axes were α = 17.2°, β = −2°, and γ = 13.2° for the first casting, and α = 35.6°, β = −4.4°, and γ = 25.4° for the second casting.

**Conclusion:**

Using the proposed approach has led to good correlation between the simulation results and the actual situation. It was feasible to use a non-radiological scanner coupled with FEM to evaluate the clubfoot treatment, which eliminates the risk of x-ray exposure for infants.

## 1 Introduction

Talipes equinovarus, also known as clubfoot, was one of the most common congenital defects in newborn infants. The occurrence was one in 1,000 live births (1). The non-surgical Ponseti treatment of clubfoot has become more popular worldwide since 1990 (2). This method was a conservative procedure using progressive manipulation and casting technique, tenotomy of the Achilles tendon, and foot bracing. Compared with the traditional surgical approach, the Ponseti method has lowered the barriers for patients in the aspects of financial and culture.

However, the application of the Ponseti method therapy was largely dependent on the surgeon's experiences, especially for the assessment of severity and evaluation of corrective manipulation procedures. The Ponseti method commonly involves 4–6 times progressive angle correcting manipulation and casting. For each manipulation, the determinants were corrective angles and forces. The distribution and level of forces were strongly related to the geometry and biomechanical properties of patients. Therefore, the evaluation of mechanical stress could avail of the manipulation determinants of the treatments.

Combining computational simulation and image processing techniques, the recent advanced assessment method has been developed with the modelling of the finite element method (FEM) (3–10) and computed tomographic (CT) scan (11–13). From the CT scan image, the morphology of bones, cartilages, ligaments, and tendons could be digitised and imported to the FEM software. The deformity of clubfoot could be accurately represented. This approach enabled the estimation of mechanical strain and stress related to the deformation of the foot, which was beneficial for the selection of treatment strategies.

FEM was a well-developed computational simulation technology, allowing the development of medical applications for various treatments and assessments based on biomechanical simulations. The mechanical strain and stress could be estimated according to the material properties and boundary loadings, which was a powerful tool for foot disease diagnosis and assessment.

The CT scan was one of the most common diagnostic radiology examinations, which uses x-rays in diagnosis. Children were more radiosensitive compared with adults due to their rapid DNA reproduction during growth. Increasing the cumulative radiation dose of a CT scan will increase the risk of leukemia and brain tumors. The threshold of cumulative CT scan dose to triple the risk of leukemia was 50 mGy. To triple the risk of brain tumor, the cumulative CT scan dose threshold was 60 mGy (14). Due to the technical and privacy issues, recording the cumulative CT scan dose of patients remains challenging (15).

The optical scanning technique was safer in pediatric diagnosis of clubfoot due to its LED-based optical light-emitting scanning and measurement techniques. The scanners do not emit any ionising radiation or high-energy lasers, which will not affect the DNA of children.

The FEM application of clubfoot assessment has not been broadly studied. The purpose of this study was to demonstrate and evaluate a new clubfoot assessment approach combining laser scanning techniques for modeling and computational simulation using FEM, which avoids the exposure to x-ray for patients and reduces the risk of radiation induced cancers.

## 2 Materials and methods

A 6-month-old male child patient was evaluated with a diagnosis of congenital clubfoot. The left foot was scanned by an LED-based optical scanner (EinScan-Pro, SHINING 3D Tech. Co., Ltd., Hangzhou, China). The usage of the patient's data has been approved by the Ethics Committee of Qingdao University. The 3D model was created through a binocular stereo vision technique. After scanning the foot, the 3D surface model was generated by the scanner in Polygon File Format (ply) or Stanford Triangle Format (stl). The FEM analysis requires 3D volume information to generate a mesh. Geomagic software (3D Systems, Inc., NC, USA) was used to close the 3D surface model generated by the scanner and convert it to a well-defined 3D volume model. The 3D volume model was imported to COMSOL (COMSOL, Inc., MA, USA) for FEM analysis. The model contains 319,916 tetrahedral mesh elements to capture the stress variation of the foot.

The specific operation process of Ponseti was as follows: (1) It was essential to conduct a comprehensive assessment of the severity of the child's foot deformities, including cavus, adduction of the forefoot, inversion of the hindfoot, and plantar flexion of the ankle joint, etc. (2) Follow the sequence of first correcting the high arch and anterior foot adduction, then the posterior foot inversion, and finally the plantar flexion of the ankle joint, gradually achieving three-dimensional correction of the deformity to avoid soft tissue injury or epiphyseal injury caused by violent correction. (3) Correction of clubfoot was accomplished by abducting the foot in supination while counterpressure was applied over the lateral aspect of the head of the talus to prevent rotation of the talus in the ankle. A well-molded plaster cast maintains the foot in an improved position. The length of the plaster cast should be from the toe to the middle and upper one-third of the thigh, maintaining a knee flexion of approximately 90°. (4) After the anterior foot was adducted and the posterior foot was turned inward, if the ankle joint cannot be passively extended to the neutral position, percutaneous Achilles tendinotomy should be performed, followed by a long leg tubular plaster cast fixation for three weeks. (5) The brace should be worn full-time (day and night) for the first 3 months after the last cast was removed. After that, the child should wear the brace for 12 h at night and 2 to 4 h in the middle of the day, for a total of 14 to 16 h during each 24 h period. This protocol continues until the child was 3 to 4 years of age. (6) It was very important to guide family members to do passive foot stretching exercises and have regular follow-ups.

The Ponseti therapy used the external fixation to apply correction forces, which act on the contact surface between the foot and the cast. In order to reflect the actual boundary condition to estimate the mechanical stress and strain induced by the angle correction, a contact model was developed. A solid was initially set to be parallel and next to the pelma at the start of the simulation. The prescribed boundary condition was selected to rotate the solid to a horizontal position, which represented the angle correction process of Ponseti. The prescribed displacement of each node could be calculated by the transformation. The transformation matrix T was

$$T=\left\{\begin{array}{ccc} \cos\;\beta \cos\;\gamma & \cos\gamma \sin\;\alpha \sin\;\beta -\cos\;\alpha \sin\;\gamma & \sin\;\alpha \sin\;\gamma +\cos\;\alpha \cos\;\gamma \sin\;\beta \\ \cos\;\beta \cos\;\gamma & \cos\;\alpha \cos\;\gamma +\sin\;\alpha \sin\;\beta \sin\;\gamma & \cos\;\alpha \sin\;\beta \sin\;\gamma -\cos\;\gamma \sin\;\gamma \\ \sin\;\beta & \cos\;\beta \sin\;\alpha & \cos\;\alpha \cos\;\beta \end{array}\right\}\;\;\;$$ (1)

where α, β, and γ denote the rotating angle along the X, Y, and Z axes, respectively (Equation 1). The relationship between the initial points P and transformed P’ could be described as

$$P^{\prime }=T\times P+P_{0}$$ (2)

where P_0_ was the coordinate of the rotating point (Equation 2). To determine the rotating angle, the scanned foot model was transformed to the standard position with the lower leg staying vertical and the center. The solid was rotated along a point P_0_ from horizontal to be parallel with a gap of less than 2 mm. The rotating angles for α, β, and γ were 40°, −5°, and 30°, respectively. The coordinate of the rotating point was selected to be the center of the ankle joint.

In this study, the contact was handled by relating a pseudo penalty force T with the material effective gap distance dg that could be expressed as

$$T=\{\begin{array}{c} -p_{n}d_{g}+p_{0}\;\;\;\;\;\;\;\;\;ifd_{g}<\frac{\;p_{0}}{\;p_{n}}\\ 0,\;\;\;\;\;\;\;\;\;\;\;\;\;\;\;\;\;\;\;\;\;\;\;\;\;\;\;\;ifd_{g}<\frac{\;p_{0}}{\;p_{n}} \end{array}$$ (3)

Where *p_n_* was the penalty factor, and *p_0_* was the pressure at zero gap (Equation 3). In this study, the *p_0_* was set to zero, and the *p_n_* was set to 0.5 MPa.

As the infant's bones were not well developed, the mechanical elastic modulus was much lower than that of adults. The mean elastic modulus was 451 MPa, and the strength was 45.4 MPa for infants younger than 12 months (16). Previous studies have shown that the maximum stress acting on the bones was 4.26 MPa for Ponseti therapy (17), which was less than 10% of the strength. The most important factor for clubfoot therapy assessment was the tension stress concentrated on the Achilles tendon. The mechanical properties of tendons were close to those of ligaments and other soft tissues. Therefore, the foot was treated as an isotropic material. The aim was to identify the relationship between the angle of correction and stretch that could be used for clinical assessment of clubfoot and therapy planning.

The material model for the infant foot was the isotropic elastic model with 0.5 MPa elastic modulus. The Poisson's ratio was set to 0.5 based on the assumption of incompressible soft tissue. The solid material was an elastic material with an elastic modulus of 100 MPa and a Poisson's ratio of 0.3.

## 3 Result and discusion

**Figure 1:**
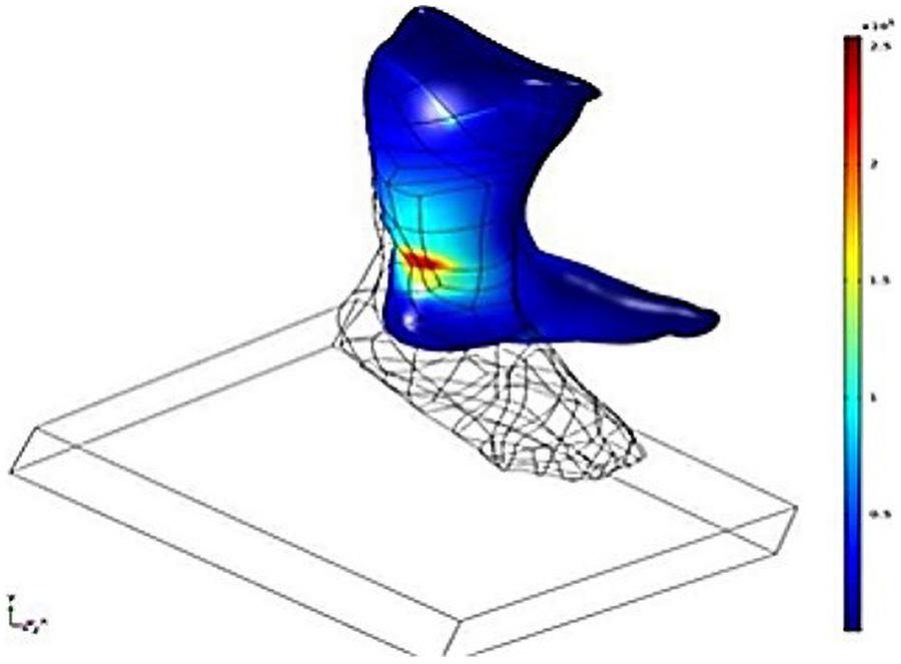
The von-Mises stress distribution and model configuration.

The major objective was to develop a new approach to identify the biomechanical stress distribution during Ponseti therapy for infant clubfoot without using x-ray-based scanning techniques. The 3D model was acquired through the LED-based optical scanner (5, 13). The advantage of this approach was that it eliminates the x-ray dosage during the assessment process of clubfoot treatment, which reduces the risk of leukemia and brain tumors induced by radiation dose. The intrinsic safety of this approach was more acceptable than CT to the patients’ families. The 3D model (18–20) acquired through optical scanning only contains external surface information. Considering that the infant tendon and ligament have close mechanical properties, the isotropic material model was applied and demonstrated a good prediction of stress concentration. In the simulation result, the highest stress concentration occurred at the Achilles tendon and the fixation contact area at the lower leg (Figure 1). The prediction of the stress concentration location coincided with the real-world situation. During the Poseti therapy, the most concerning factor that restrains the correction angle was the stress concentration at the foot. The simulation with isotropic material property has demonstrated the capability to represent real-world stress distribution, which proves that using a non-radiation-based optical light scanning technique was adequate for infant clubfoot assessment. This approach was effective and safer than CT-based assessment, which could eliminate the radiation exposure and reduce the risk of leukemia and brain tumors (21).

FEM exhibited a close intrinsic relationship with the Ponseti method for treating clubfoot. Its core lied in constructing three-dimensional foot models to quantify the mechanical properties of bones and soft tissues, thereby providing objective data support for optimizing the Ponseti method. Pre-treatment involved constructing personalized finite element models to achieve a quantitative biomechanical assessment of clubfoot. During treatment, simulated foot mechanics under varying orthotic parameters enables precise optimization of critical factors such as cast correction angles and fixation cycles, providing real-time guidance for accurate Ponseti implementation. Post-treatment evaluation of foot mechanics facilitates optimization of brace design and parameter settings, enhancing the effectiveness of maintenance therapy.

**Figure 2:**
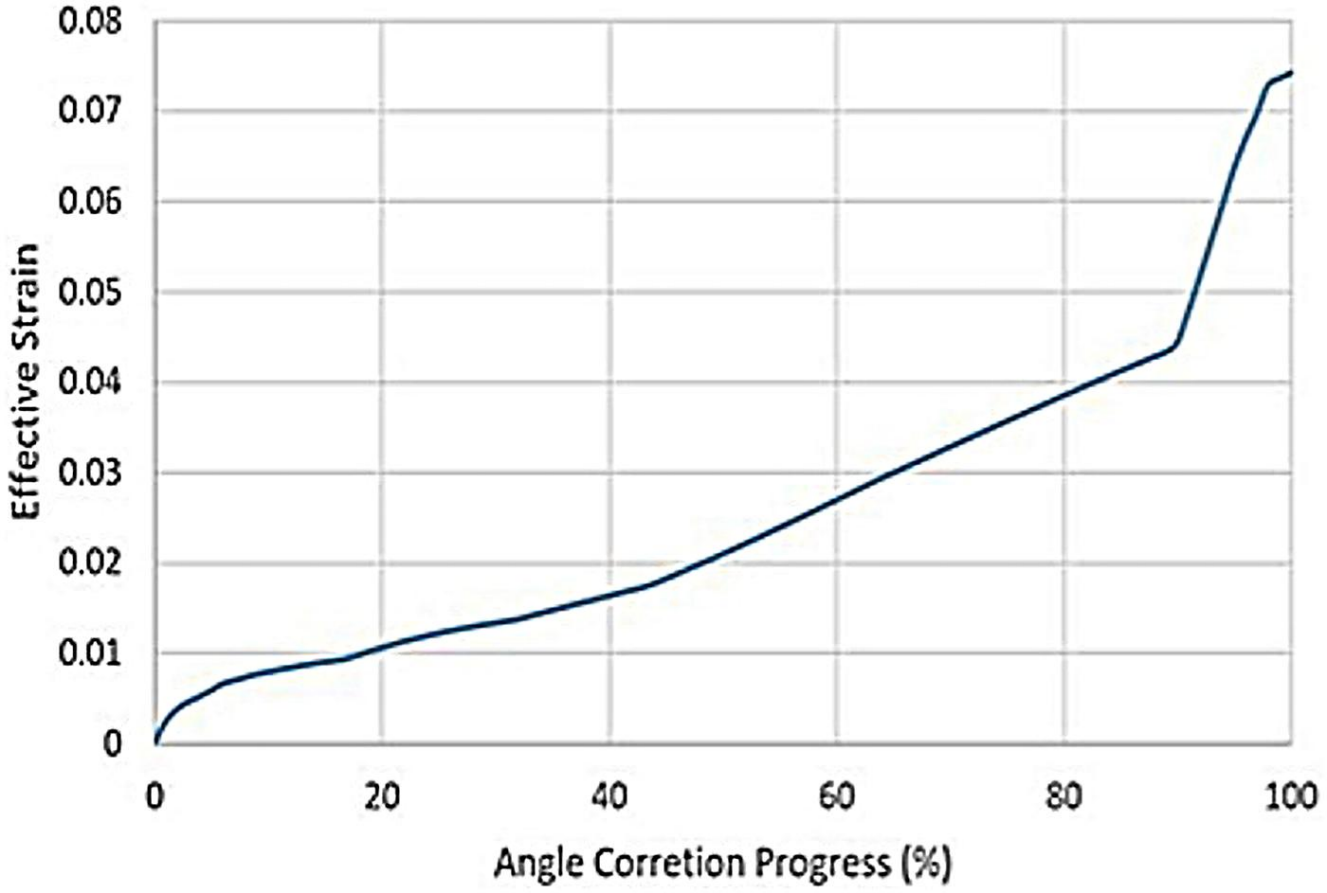
The effective strain vs. angle correction progress. The maximum strain was less than the calculated rupture strain of 0.087.

**Figure 3:**
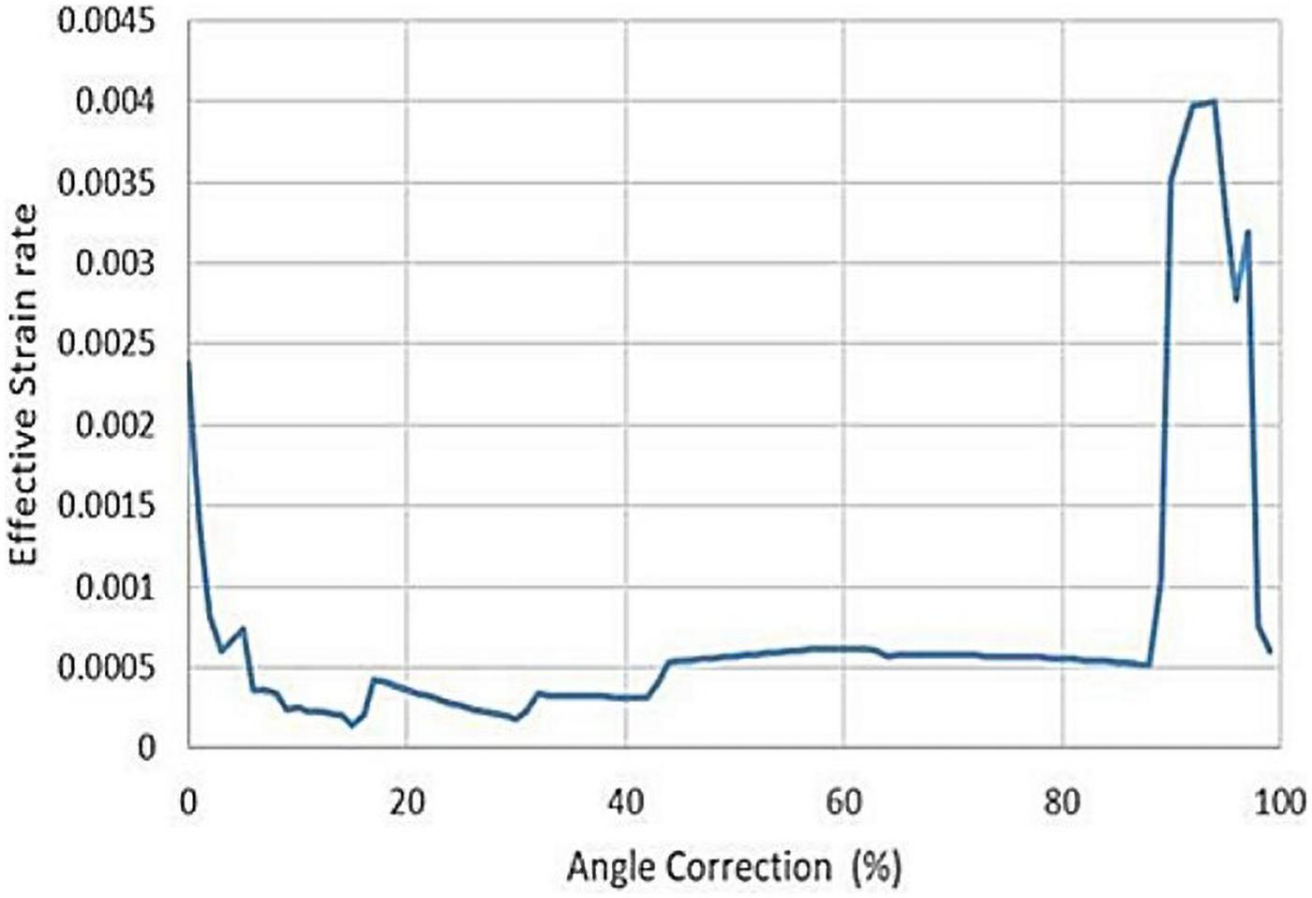
The pseudo strain rate vs. angle correction. Three-stage strain rate variation was observed, which coincided with the three-stage casting of Posenti therapy.

The tendon stretch rupture will cause an irregular fracture surface due to the complexity of the tendon structure, which affects the healing process. The Achilles tendon rupture during Ponseti therapy should be avoided. It was critical that the tendon rupture criteria were not met during the process. To evaluate the risk of rupture, the maximum effective stress or strain could be compared with the rupture stress or strain. The mechanical rupture stress of the infant Achilles tendon was difficult to obtain due to the limited research and doners. The Younge's modulus and rupture stress of adults Achilles tendon were measured *in vitro* (22). The average rupture strain could be estimated by

$$\varepsilon_{r}=\sigma_{r}/E$$ (4)

where *ε_r_* was the rupture strain, *σ_r_* was the rupture stress, and *E* was the Younge's modulus, respectively (Equation 4). The values of *E* = 816 MPa and *σ_r_* = 71 MPa were reported in previous research (22). As the Younge's modulus of the Achilles tendon varied significantly according to age, it was more reasonable to use *ε_r_* as the rupture criterion with the assumption that the rupture strain was not sensitive to the strength of the tendon. The calculated *ε_r_* was 0.087, which was used to evaluate the maximum allowable angle correction for each casting. Comparing the *ε_r_* with the effective strain, the correction angle should not cause excessive strain around the Achilles tendon. The relationship between the effective strain and the angle correction process was plotted in Figure 2. The maximum effective strain was 0.074, which was lower than the rupture strain of 0.087. The effective strain increased with the correction angle nonlinearly. In order to analyse the development of the effective strain, a pseudo strain rate $\varepsilon \dot{s}$ was introduced as the derivative of effective strain over angle correction percentage, which developed in three stages, as shown in Figure 3. In the initial stage from 0 to 40%, the pseudo strain rate started from 0.00237 and dropped to a steady value around 0.0003. The pseudo strain rate increased and remained at a steady value around 0.0006 until the angle correction of 87%. In the last stage, where the angle correction was above 87%, the pseudo strain rate increased significantly to 0.004. The Ponseti therapy was recommended to correct the angle in three stages with three castings (23–26). The Achilles tendon needed to be amputated in the last stage to reduce the stretch. The simulated pseudo strain rate also indicated three stages of development. Especially, the effective strain increased significantly in the last stage, which coincided with the Ponseti therapy process. The current study provided an approach of quantitative explanation of the appropriate angle for Achilles tendon amputation. The results also suggested that 40% and 87% were the favourable angle corrections for intermediate casting, in order to reduce the risk of tendon rupture.

**Figure 4:**
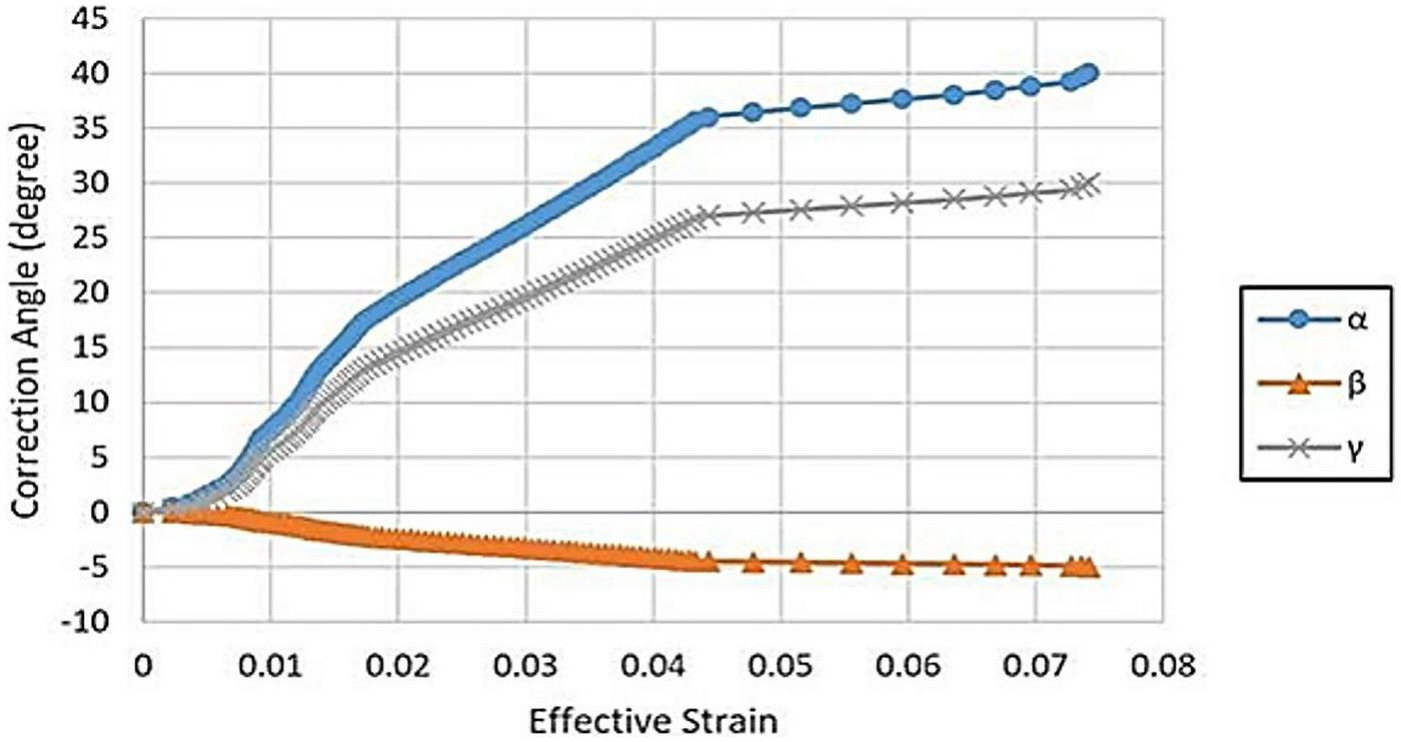
The rotation angles vs. effective strain. α, β and γ are the rotation angles around three axis of coronal, axial and sagittal plane normal.

The correction angle could be broken down to three components in spatial coordinates, namely the rotation angles α, β, and γ around three axes of the coronal, axial, and sagittal plane normal. The relationship between the effective strain and rotation angles was plotted in Figure 4. The three-stage development of the curves could be observed. The suggested intermediate casting angles were α = 17.2°, β = −2°, and γ = 13.2° for the first casting, and α = 35.6°, β = −4.4°, and γ = 25.4° for the second casting.

The study took a single case of clubfoot in children as the entry point, which had limitations such as a small sample size and limited extrapolation of the conclusion. The following research will further verify the reliability and applicability of FEM in the diagnosis and treatment of this disease by increasing the sample size.

## 4 Conclusion

The current study developed an approach for clubfoot assessment, which used an optical-light-based scanner to acquire the geometry and an FEM simulation to evaluate the correction angle of the Ponseti therapy process. This method eliminated the x-ray exposure of the patients, which reduced the risk of leukemia and brain tumors. The simulation result demonstrated good correlation with the real-world situation, which proved the feasibility and accuracy of this approach. The effective strain and the pseudo strain rate were calculated to assess the risk of Achilles tendon rupture, as well as providing recommended intermediate casting angles. The implementation of this approach could lead to a safer and quantitative assessment of customised clubfoot treatment.

## Data Availability

The original contributions presented in the study are included in the article/Supplementary Material, further inquiries can be directed to the corresponding author.
